# A novel method for the identification of synchronization effects in multichannel ECoG with an application to epilepsy

**DOI:** 10.1007/s00422-013-0552-8

**Published:** 2013-02-22

**Authors:** A. Graef, M. Hartmann, C. Flamm, C. Baumgartner, M. Deistler, T. Kluge

**Affiliations:** 1 Institute for Mathematical Methods in Economics, Vienna University of Technology, Vienna, Austria; 2 Department Safety & Security, AIT Austrian Institute of Technology GmbH, Vienna, Austria; 3 Karl Landsteiner Institute for Clinical Epilepsy Research and Cognitive Neurology, General Hospital Hietzing with Neurological Center Rosenhügel, Vienna, Austria

**Keywords:** Epilepsy, ECoG, Partial directed coherence, Synchronization, Dynamic input channel selection

## Abstract

In this paper, we present a novel method for the identification of synchronization effects in multichannel electrocorticograms (ECoG). Based on autoregressive modeling, we define a dependency measure termed extrinsic-to-intrinsic power ratio (EIPR) which quantifies directed coupling effects in the time domain. Hereby, a dynamic input channel selection algorithm assures the estimation of the model parameters despite the strong spatial correlation among the high number of involved ECoG channels. We compare EIPR to the partial directed coherence, show its ability to indicate Granger causality and successfully validate a signal model. Applying EIPR to ictal ECoG data of patients suffering from temporal lobe epilepsy allows us to identify the electrodes of the seizure onset zone. The results obtained by the proposed method are in good accordance with the clinical findings.

## Introduction

### Medical background

Epilepsy is a chronic neurological disorder with a prevalence of ${\sim }$0.7 % (Hirtz et al. 2007) and is characterized by the recurrence of unprovoked and unpredictable seizures. The seizures can be described as a frequently recurring temporary occurrence of hyper-synchronous activity within relatively large areas of the cortex that severely disturbs normal brain function. Often, the pathological synchronous activity (synchronizations) starts at a small, localized brain area within the gray matter and then spreads to its immediate vicinity recruiting more and more parts of the neural network. In the case of generalized epilepsy, the synchronous neural firing can affect the whole cortex, in the case of focal epilepsy only parts of it, the so-called epileptogenic area is affected (Baumgartner 2001).

About one-third of epilepsy patients suffer from therapy-resistant seizures, i.e., seizures cannot be controlled with anti-epileptic drugs (Engel 1996). Epilepsy surgery has become a valuable treatment option for some of these patients rendering 70–80 % of them seizure-free and providing them with the opportunity for a satisfactory life (Clusmann et al. 2002). The aim of epilepsy surgery is removal of the epileptogenic brain region, whereby a complete removal of the epileptogenic zone is essential in order to abolish the seizures. At the same time essential brain regions like, e.g., primary motor and sensory cortex, as well as brain areas supporting language and memory functions have to be spared in order to avoid neurological deficits caused by the surgery (Lüders 1992). Thus, the exact localization of the epileptogenic zone and of essential brain regions are crucial for the successful surgical treatment of seizures which can only be accomplished during a thorough presurgical evaluation. This examination comprises prolonged video-EEG recording, high resolution brain imaging, and neuropsychological tests.

Especially in patients with seizures arising adjacent to essential brain regions, invasive *electroencephalogram (EEG)* recordings with chronically indwelling subdural strip and grid electrodes (termed*electrocorticogram, ECoG*) or depth electrodes have to be applied in order to increase spatial resolution (Behrens et al. 1994; Zumsteg and Wieser 2000). However, even with these invasive techniques the epileptogenic zone cannot be localized adequately in about 20 % of patients (Pondal-Sordo et al. 2007). Thus, these patients cannot be offered a surgical therapy and the electrodes have to be removed without resective surgery.

In order to localize the epileptic zone and analyze the propagation of the seizure, visual inspection of raw ECoG data is performed by clinicians. This is a difficult and time-consuming task, but still regarded as gold standard (compare Götz-Trabert et al. 2008; Jenssen et al. 2011 for two recent studies). Therefore improving the analysis of the initial seizure propagation using mathematical models is clinically desired: Identifying the spatio-temporal dependencies in ictal ECoG recordings could fulfill this task.

### Technical background

For localizing the onset zone of the epileptic activity, we want to visualize coupling effects of the multivariate ECoG signal. For this purpose, we calculate dependency measures and depict them in a graph, whose vertices represent the components of the signal, and edges indicate dependencies different from zero. This approach yields an intuitive graphical representation of coupling effects in multivariate time series (cf. e.g., Dahlhaus 2000; Dahlhaus and Eichler 2003; Eichler 2006a).

A wide variety of different coupling indicators for neurophysiological data has been published: Pereda et al. (2005) give an overview of common nonlinear approaches to the analysis of neurophysiological data. Among them, similarity measures have recently been used by Hedge et al. (2005) for tracking spatio-temporal dependencies in ictal ECoG recordings.

Winterhalder et al. (2005) discuss dependency measures in the linear framework of autoregressive (AR) modeling. Two important directed coupling indicators are the *directed transfer function (DTF)* and the *partial directed coherence (PDC)*, both distinguishing between source and target by indicating a direction of the dependency. DTF and PDC are based on a common linear approach to EEG analysis in the literature (Franaszczuk et al. 1985; Sanei and Chambers 2007; Tong and Thakor 2009): To model the ECoG signal as a multivariate AR process. Once the AR model has been identified, spectral properties directly follow (Marple 1987). The spectrum is the basis for the measurement of linear couplings in the frequency domain, which reveal relations between electrodes. These inter-dependencies are interpreted as indications for epileptic synchronous activity.

The *DTF* was proposed by Kaminski and Blinowska (1991) for quantifying dependencies in neural signals. However, when Franaszczuk et al. (1994), Franaszczuk and Bergery (1998), and Ge et al. (2007) used DTF for epileptic seizure analysis, a manual selection of narrow frequency bands always had to be performed in order to achieve satisfying results.

Wilke et al. (2008) proposed a time-variant version of DTF for epileptic EEG analysis, which shows promising first results. Recently, Kim et al. (2010) combined the usage of DTF with a spatio-temporal source localization algorithm in order to analyze the propagation of epileptic activity in ECoG signals.

As DTF is not capable of distinguishing between direct and indirect influences, Korzeniewska et al. (2003) proposed an extension termed *direct directed transfer function (dDTF)*, which only indicates direct coupling effects and ignores indirect ones.

The *PDC* is another prominent coupling indicator realizing this distinction between direct and indirect influences. It is directly based on the Fourier-transformed coefficients of the AR model. As the relation between Granger causality—a concept of directed dependency developed by Granger (1969)—and DTF is controversial (Eichler 2006b; Kaminski et al. 2001), Baccala and Sameshima (2001) proposed PDC in order to provide a frequency-domain picture of Granger causality.

In the last years, there has been an increasing interest in this dependency measure: PDC has often been applied to the analysis of neural interactions (e.g., Sameshima and Baccala 1999; Astolfi et al. 2005), and various extensions to the initial form of PDC have been proposed: First, Baccala et al. (2007) generalized their original definition in order to provide a scale-invariant form. Recently, Takahashi et al. (2010) established a link between a slightly redefined form of PDC and the mutual information rate (MIR), an information-theoretic consideration of couplings. Finally, Faes and Nollo (2010) considered an extended version of PDC for the modeling of instantaneous dependencies between EEG channels.

### Motivation

Our aim is to analyze synchronization effects in multichannel ECoG data of epileptic patients and to identify these coupling effects with a high degree of automatism: Unlike the methods mentioned above, we neither want to manually preselect ECoG input channels nor explicitly consider specific frequency bands. Our contributions to epileptic seizure analysis are twofold.

In order to avoid numerical problems resulting from the high number of ECoG channels, we suggest an automatic channel selection prior to computing dependency measures. This idea is detailed in Sect. 2.4.

Furthermore, we propose a different approach to the identification of synchronous activity: Contrary to a spectral analysis, as it is performed by DTF or PDC, we directly consider couplings in the time domain. In order to assure a (neuro)physiological interpretation of our methodology, we search for a coupling indicator with a clear physical interpretability. For this purpose, we introduce a novel dependency measure termed *extrinsic-to-intrinsic power ratio (EIPR)* initially defined by Hartmann et al. (2008), which is discussed in Sect. 2.5.

The method is tested on neural signals for the localization of the epileptic seizure onset zone. A method based on these results might be used in the future in order to provide neurologists with a tool yielding a seizure onset zone localization which supports them in a clinical environment.

## Materials and methods

### Definitions and assumptions

In our analysis of multichannel ECoG data, we deal with multivariate signals $\mathbf x [t]$ ($t\in \mathbb Z$ denoting the time index), which consist of $K$ real-valued components $x_{k}[t],\, k=1,\ldots ,K$. We call the components $x_{k}[t]$ *channels*, representing sampled ECoG recordings at a sampling frequency $f_\mathrm{s}$. Fourier-transformed signals are denoted by their respective capital letter [e.g., $\mathbf x [t]$ becomes $\mathbf X (f)$, see Marple (1987) for details].

In order to process the recorded data, we use a window of length $T_\mathrm{win}$, therefore containing $N_\mathrm{win}=T_\mathrm{win}\cdot f_\mathrm{s}$ samples. Within this data window, we assume the channels to be zero-mean and stationary, i.e., we have time-invariance of the first- and second-order statistics. In the remainder of this paper, we will refer to time indices relative to a data window by $n$ (therefore we have $n=1,\ldots ,N_\mathrm{win}$ in each window).

We use common mathematical abbreviations: We denote the expectation by $\mathbb{E }\left\{ \cdot \right\}$ and the variance by $\mathbb{V }\left\{ \cdot \right\}$. Furthermore, the complex unit is symbolized by $i$.

### Autoregressive model

For an introduction to multivariate time series analysis, in particular to multivariate AR modeling, we refer to (Lütkepohl 2007) and (Hannan and Deistler 1988).

The proposed method starts with the definition of a stable multivariate AR model of order $p$, defined by

1 $$\begin{aligned} \mathbf{x }[n]=\sum _{j=1}^{p}\mathbf{A }[j]\,\mathbf{x }[n-j]+\varepsilon [n], \end{aligned}$$

where $\epsilon [n]$ is white noise with regular covariance matrix $\Sigma _{\epsilon }$. This model is decomposed component-wise into the separated contributions of all channels: We define the *partial contribution* $\mu _{k,l}[n]$ as

2 $$\begin{aligned} \mu _{k,l}[n]\triangleq \sum _{j=1}^{p}A_{k,l}[j]\, x_{l}[n-j] \end{aligned}$$

with $A_{k,l}[j]$ the (k,l)-element of the coefficient matrix $\mathbf A [j]$ in (1). This allows to write the AR model (1) for each channel $x_{k}[n],\, k=1,\ldots ,K$ as

$$\begin{aligned} x_{k}[n]=\mu _{k,k}[n]+\sum _{l\ne k}\mu _{k,l}[n]+\varepsilon _{k}[n]. \end{aligned}$$

In order to shrink the regression model, we only consider partial contribution terms $\mu _{k,l}[n]$ which significantly differ from zero. The explicit choice of inputs yields a model of the form

3 $$\begin{aligned} x_{k}[n]=\mu _{k,k}[n]+\sum _{l{\in \mathbb L }_{k}}\mu _{k,l}[n]+\tilde{\varepsilon }_{k}[n],\quad k=1,\ldots ,K. \end{aligned}$$

Here $\mathbb{L }_{k}$ is an *extrinsic channel set*, which can be a subset of $\{1,\ldots ,K\}\backslash \{k\}$, allowing for a reduction of the number of parameters of the AR model. A strategy for such an order reduction is proposed in Sect. 2.4.

Thus each $\mu _{k,l}[n]$ in Eq. (3) reflects the contribution from (the past of) the respective channel $x_{l}[n]$ to channel $x_{k}[n]$. As we differentiate between the channel $x_{k}[n]$ and the other $x_{l}[n],\, l\ne k$ in Eq. (3), we introduce the following specification: For $k=l$, we call $\mu _{k,k}[n]$ is the *intrinsic contribution*; for $k\ne l$, $\mu _{k,l}[n]$ is the *partial extrinsic contribution*. As the term $\sum _{l\in \mathbb L _{k}}\mu _{k,l}[n]$ in Eq. (3) is the sum of all partial extrinsic contributions, it symbolizes the total amount of inflow to the channel $x_{k}[n]$ and is therefore denoted by *total extrinsic contribution*.

A more general definition of the autoregression in the partial contribution term (2), as proposed by Hartmann et al. (2008), would allow a more flexible lag usage, e.g., permitting non-causal modeling.

### Solution of the normal equations

Under the assumption of short-term stationarity, the solution of the normal equations (within the data window) yields the estimated model coefficients $A_{k,l}[j]$ in the sense of ordinary-least-squares (OLS). Second-order statistics needed for their solution have to be estimated from the data. For this reason, it is important that the length of the data window is chosen neither too short nor too long. An appropriate choice has to establish a good trade-off between estimation errors due to instationarity and inaccuracy due to a too small number of samples.

In particular, in case of neural data such as ECoG recordings their highly instationary character requires the use of short data windows.

### Dynamic input channel selection

The estimation of the model coefficients $A_{k,l}[j]$ in the normal equations poses numerical problems, as we deal with a large number of ECoG channels which are highly correlated both in time and in the cross-sectional dimension. In order to avoid this situation, the idea is therefore to automatically reduce the number of channels in a subset containing all information important for the regression.

For this reason, we introduced the extrinsic channel set $\mathbb L _{k}$ in Eq. (3), which defines—per channel $x_{k}[n]$—the $x_{l}[n]$ relevant for the AR model. The advantage arising from this approach is that we do not have to choose $\mathbb L _{k}=\{1 \ldots K\}\backslash \{k\}$ (as it would be the case in the AR model (1), but can shrink it to a reduced set of channels. We only consider the channels $\{x_{k},\, x_{l}:l\in \mathbb L _{k}\}$, and this selection assures that the estimation of the model coefficients yields numerically stable results: The correlation matrix of the small subsystem is well conditioned and can be inverted without further numerical problems.

**Fig. 1:**
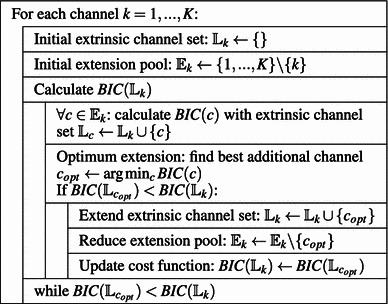
Automatic channel selection algorithm. The bottom–up construction of the extrinsic channel set $\mathbb L _{k}$ is given in pseudo code

We propose an iterative procedure for an automatic selection of an extrinsic channel set $\mathbb L _{k}$ for each $x_{k}$, which is described in pseudo code in Fig. 1.

The main idea is to iteratively add channels in a bottom–up fashion until the *Bayesian information criterion (BIC)* 1 is minimized: In this context, it is defined as (Penm and Terrell 1982)

4 $$\begin{aligned} \text{ BIC}(\mathbb{L }_{k})\triangleq \ln \, S_\mathrm{err}(\mathbb{L }_{k}\cup \{k\})+\frac{M\,\ln N_\mathrm{win}}{N_\mathrm{win}}, \end{aligned}$$

where

$$\begin{aligned} S_\mathrm{err}=\sum _{n=1}^{N_\mathrm{win}}\left(\tilde{\varepsilon }_{k}[n]\right)^{2} \end{aligned}$$

is the residual sum of squares and

$$\begin{aligned} M=\left(\sum _{s=1}^{K}\delta _{s}(\mathbb L _{k})+1\right)\cdot p \end{aligned}$$

with

$$\begin{aligned} \delta _{s}(\mathbb L _{k})= {\left\{ \begin{array}{ll} 1&s \in \mathbb{L }_{k}\\ 0&s \notin \mathbb{L }_{k} \end{array}\right.}. \end{aligned}$$

Hence $M=(\dim \,\mathbb L _{k}+1)\cdot p$ is the total number of parameters to be estimated.

Using this criterion our algorithm works as follows: We start with an empty extrinsic channel set $\mathbb L _{k}$. Then, we add the channel $x_{l}[n],\, l\ne k$ of the $(K-1)$ other ones which is best in the sense that it leads to the smallest BIC value (4). In the next step, we again select the “best” out of the remaining ones and so forth till we cannot decrease the value of the bracket expression in criterion (4) any more by adding channels. This (local) minimum determines the extrinsic channel set $\mathbb L _{k}$ to be used for coefficient estimation. Coefficients $A_{k,l}[j]$ of channels $x_{l}[n]$ which were not selected by this iterative procedure are set to zero.

We expect the algorithm to select extrinsic channels which contribute significantly to the explanation of the respective intrinsic channel. We will illustrate this behavior in Sect. 3.2 in detail.

Note that the bottom–up approach of our proposed algorithm is similar to the *An algorithm* published by An and Gu (1989), which is however limited to a regression model without any temporal lags.

### Partial extrinsic power

The aim of the proposed method is to identify directed dependencies of the multivariate signal $\mathbf x [n]$, which are expected to indicate synchronization and coupling effects of brain regions during epileptic seizures. For similar problems, numerous alternative measures based on a spectral analysis have been proposed in the literature (compare Sect. 1.2). However, instead of regarding spectral properties of the AR model (3), we propose to directly consider the partial contribution term (2) in order to gain information on the influence of channel $x_{l}[n]$ to channel $x_{k}[n]$.

The variance of the partial contribution term $\mu _{k,l}[n]$ can be written as, using (2),

5 $$\begin{aligned} \mathbb{V }\left\{ \mu _{k,l}[n]\right\}&= \mathbb{E }\left\{ \mu _{k,l}[n]\,\mu _{k,l}[n]\right\} \nonumber \\&= \sum _{j=1}^{p}\sum _{j^{\prime }=1}^{p}A_{k,l}[j]\, r_{x_{l}}[j-j^{\prime }]\, A_{k,l}[j^{\prime }], \end{aligned}$$

where $r_{x_{l}}[s]=\mathbb{E }\left\{ x_{l}[n+s]\, x_{l}[n]\right\}$ is the autocorrelation function of channel $x_{l}[n]$.

The partial contribution term $\mu _{k,l}[n]$ represents the directed influence of channel $x_{l}$ onto $x_{k}$ by construction, see model (1). Its variance is a natural measure of the strength of the influence from $x_{l}$ onto $x_{k}$. Therefore, we expect it to gain importance in ictal periods due to the increased synchronous activity mentioned in Sect. 1.1. For $k=l$, we speak about the *intrinsic* *power*, for $k\ne l$ about the *partial extrinsic power*.

Note that although the sum of all partial extrinsic contributions $\mu _{k,l}[n]$ gives the total extrinsic contribution (i.e., the inflow from all channels $x_{l}[n]$ onto channel $x_{k}[n]$, $k\ne l$), the sum of all partial extrinsic power terms $\mathbb V \left\{ \mu _{k,l}\right\}$ does not equal the total extrinsic power $\mathbb V \left\{ \sum _{l\in \mathbb L _{k}}\mu _{k,l}[n]\right\}$, unless all cross-correlations are zero.

Thus, considering the variance (5) of the respective partial contribution term for all channel combinations $k, l=1,\ldots ,K$, we expect to obtain an indication for the directed coupling of each channel $x_{l}[n]$ onto each channel $x_{k}[n]$ in scalar form. We can finally represent this $K\times K$ matrix of coupling information in a graph.

### Extrinsic-to-intrinsic power ratio (EIPR)

A problem with the variance (5) of the partial contribution term is its scale-dependence. It is desirable to normalize this measure appropriately such that it is independent of the signal power.

It is not obvious how to perform this normalization. One could, for example, normalize with respect to all target channels, as done by the PDC, as introduced below. However, this approach renders the measure dependent of all channels involved in the regression (cf. Schelter et al. 2009). This will be detailed in the next subsection.

This motivates our search for an alternative normalization which is not affected by this kind of limitation. We propose to use the *EIPR*

6 $$\begin{aligned} \eta _{k,l}^{2} \triangleq \frac{\mathbb{V }\left\{ \mu _{k,l}[n]\right\} }{\mathbb{V }{\left\{ \mu _{k,k}[n]\right\} }}\,, \end{aligned}$$

which was initially defined by Hartmann et al. (2008). We assume that the variance of the intrinsic contribution term in the denominator in (6) is bounded below by a positive constant^2^. This assumption was fulfilled in the considered ECoG recordings (Graef 2008).

EIPR defined in this way quantifies coupling effects of channel pairs $(x_{k},\, x_{l})$, taking large values for large partial extrinsic variance and small intrinsic variance. This is the case when channel $x_{l}$ contributes significant information to the explanation of channel $x_{k}$. On the other hand, EIPR shows only small values for weak influence of $x_{l}$ to $x_{k}$.

### Comparison of EIPR and PDC

The PDC is based on the Fourier-transformed AR model coefficients (Baccala and Sameshima 2001)

7 $$\begin{aligned} \pi _{k,l}^{2}(f)\triangleq \frac{\left|\widetilde{A}_{k,l}(f)\right|^{2}}{\sum _{n=1}^{K}\left|\widetilde{A}_{n,l}(f)\right|^{2}}. \end{aligned}$$

For frequency $f, \widetilde{\mathbf{A }}(f)\in \mathbb{C }^{K\times K}$ is obtained as

$$\begin{aligned} \widetilde{\mathbf{A }}(f)\triangleq \mathbf I -\sum _{j=1}^{p}\mathbf A [p]\, e^{-2i\pi fj}=\mathbf{I }-\mathbf{A }(f), \end{aligned}$$

where $\mathbf{A }[s]$ are the coefficients of the multivariate AR model (1), and $\mathbf{I }$ denotes the identity matrix.

As mentioned in Sect. 1.2, PDC provides a “frequency-domain picture” of Granger causality: In particular $\pi _{k,l}(f)=0\;\forall f$ implies that channel $x_{l}[n]$ does *not* Granger-cause channel $x_{k}[n]$; compare (Baccala and Sameshima 2001).

**Fig. 2:**
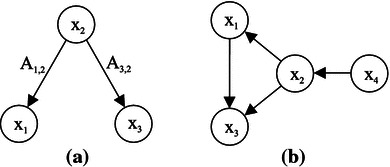
Dependency graphs of AR models. **a** illustrates a normalization problem of the PDC as discussed in Sect. 2.6, $A_{3,2}$ affects $\pi _{1,2}^{2}$. **b** shows the dependence structure of signal model (13) used for the assessment of EIPR

One important difference between EIPR and PDC is in their respective normalization. As mentioned in the previous subsection, PDC is normalized with respect to all target channels which renders the measure dependent of all channels involved in the regression (cf. Schelter et al. 2009). For an illustration consider any arbitrary three-dimensional AR model showing the dependencies depicted in Fig. 2a. When we study the coupling between two specific channels of our AR system, say $x_{2}$ and $x_{1}$, PDC is influenced by channel $x_{3}$, which is not of direct interest to us. This is easily seen from the denominator of PDC: Changing the value of the AR model coefficient $A_{3,2}$ affects the denominator of PDC

$$\begin{aligned} \pi _{1,2}^{2}(f)=\frac{\left|A_{1,2}(f)\right|^{2}}{\left|A_{1,2}(f)\right|^{2}+\left|A_{2,2}(f)\right|^{2}+\left|A_{3,2}(f)\right|^{2}}. \end{aligned}$$

Normalizing with respect to all source channels rather than to all target channels, as proposed by Schelter et al. (2009), causes similar problems.

A particular situation where a normalization either to all source or all target channels involved may lead to misleading interpretations is as follows: Assume that a seizure focus is located in the middle below three electrodes. The signals recorded depend of course on their exact position on the cortex. When studying the brain activity between two of them, our result is influenced by the position of the third electrode, which we cannot adapt to our needs (as it is implanted).

EIPR avoids this problem, as its denominator is only based on the statistics of the intrinsic (currently regarded) channel, compare (Graef et al. 2009).

Furthermore, it is interesting to note that the variance (5) of the partial contribution term is closely linked to PDC. Let us write this variance (5) as integral

8 $$\begin{aligned} \mathbb{V }\left\{ \mu _{k,l}[n]\right\} =\int _{f}S_{\mu _{k,l}}(f)\,\text{ d}f, \end{aligned}$$

where $S_{\mu _{k,l}}(f)$ denotes the spectral density of the partial contribution term $\mu _{k,l}[n]$. By transforming the partial contribution term (2) into the frequency domain, i.e.

$$\begin{aligned} M_{k,l}(f)=A_{k,l}(f)\, X_{l}(f), \end{aligned}$$

we obtain its spectral density

9 $$\begin{aligned} S_{\mu _{k,l}}(f)=\left|A_{k,l}(f)\right|^{2}S_{x_{l}}(f). \end{aligned}$$

Substituting expression (9) into the spectral representation (8), we obtain the representation

10 $$\begin{aligned} \mathbb{V }\left\{ \mu _{k,l}[n]\right\} =\int _{f}\left|\widetilde{A}_{k,l}(f)\right|^{2}S_{x_{l}}(f)\, \text{ d}f,\quad k\ne l \end{aligned}$$

of the variance of the partial contribution term $\mu _{k,l}[n]$ [cf. (5) for its definition].

Hence, under the assumption that $S_{x_{l}}(f)>0$ the left-hand side of (10) is zero if and only if PDC $\pi _{k,l}^{2}(f)=0,\, k\ne l$ for all frequencies $f$. Thus, under this assumption $x_{l}[n]$ being Granger-causal for $x_{k}[n]$ is equivalent to $\mathbb V \left\{ \mu _{k,l}[n]\right\} >0$. In particular EIPR vanishes for Granger non-causality.

Let us finally compare PDC and EIPR in the spectral domain, which underlines the reflections regarding normalization. When expressing EIPR (6) by the spectral densities of the partial contribution terms and using expression (9), we obtain

11 $$\begin{aligned} \eta _{k,l}^{2}=\frac{\int _{f}\,\left|A_{k,l}(f)\right|^{2}S_{x_{l}}(f)\, \text{ d}f}{\int _{f}\,\left|A_{k,k}(f)\right|^{2}S_{x_{k}}(f)\, \text{ d}f}=\frac{\int _{f}\, S_{\mu _{k,l}}(f)\, \text{ d}f}{\int _{f}\, S_{\mu _{k,k}}(f)\, \text{ d}f}. \end{aligned}$$

If we represent the PDC (7) by means of expression (9), we obtain

12 $$\begin{aligned} \pi _{k,l}^{2}(f)=\frac{\left|A_{k,l}(f)\right|^{2}S_{x_{l}}(f)}{\sum _{n=1}^{K}\left|A_{n,l}(f)\right|^{2}S_{x_{l}}(f)}=\frac{S_{\mu _{k,l}}(f)}{\sum _{n=1}^{K}S_{\mu _{n,l}}(f)}.\nonumber \\ \end{aligned}$$

### Signal model

As a test case for EIPR and the channel selection algorithm, we consider a simulation based on an example proposed by Winterhalder et al. (2005). This is an AR system of order $p=5$

13 $$\begin{aligned} \left\{ \begin{array}{l} x_{1}[n]= 0.8\, x_{1}[n-1]+0.65\, x_{2}[t-4]+\varepsilon _{1}[n]\\ x_{2}[n]= 0.6\, x_{2}[n-1]+0.6\, x_{4}[n-5]+\varepsilon _{2}[n]\\ x_{3}[n]\!=\! 0.5\, x_{3}[n\!-\!3]\!-\!0.6\, x_{1}[n\!-\!1]\!+\!\cdots \!+\!0.4\, x_{2}[n\!-\!4]\!+\!\varepsilon _{3}[n]\\ x_{4}[n] = 1.2\, x_{4}[n-1]-0.7\, x_{4}[n-2]+\varepsilon _{4}[n] \end{array}\right. \end{aligned}$$

with the covariance matrix of the noise set to identity. We simulate 100 s assuming a sampling frequency of $f_\mathrm{s}=128$ Hz (for consistency with the ECoG data, compare the next subsection). Note that in this artificial case we process the stationary five-dimensional signal in one single data window of length $N_\mathrm{win}=12{,}800$. The imposed dependency paths of the AR model (13) are shown in Fig. 2b. This structure was successfully retrieved by application of PDC; compare (Winterhalder et al. 2005).

As it is unlikely in applications that one observes values of EIPR exactly matching zero, one has to statistically test whether values of EIPR are significantly different from zero. As no exact distribution of EIPR is available yet, we make use of bootstrapping in order to numerically derive significance thresholds. The idea of the so-called surrogate data method (see e.g., Kaminski et al. (2001) for an application to DTF) is to resample the original data independently for each channel $x_{k}[n]$ for $N=100$ times, thus destroying the inter-channel dependence structure. This repetition gives empirical distributions of each EIPR $\eta _{k,l}^{2}$ under the null-hypothesis of non-causality ($\mathcal H _{0}:\,\mu _{k,l}=0$), namely $N$ realizations of each $\tilde{\eta }_{k,l}^{2}$. Here, we use the *leave one-out method (LOOM)* introduced by Schlögl and Supp (2006) for the re-sampling process and subsequent statistical *t* test, as it yields reliable results in causal analysis (cf. Florin et al. 2011 for a comparative study). EIPR values $\eta _{k,l}^{2}$ significantly indicate dependency if

14 $$\begin{aligned} \eta _{k,l}^{2}>-t_{N-1;\alpha }\,\frac{\hat{\sigma }(\tilde{\eta }_{k,l}^{2})}{\sqrt{N}}+\overline{\tilde{\eta }_{k,l}^{2}}, \end{aligned}$$

where $\overline{\cdot }$ denotes the empirical mean and $\hat{\sigma }(\cdot )$ the empirical standard deviation of the EIPR values $\tilde{\eta }_{k,l}^{2}$ based on the re-sampled data. $t_{N-1;\alpha }$ is the quantile of the Student distribution with $N-1=99$ degrees of freedom and $\alpha =1-0.99=0.01$.

### ECoG data

The ECoG data in this study are taken from four female patients between 31 and 61 years of age suffering from therapy-resistant temporal lobe epilepsy. The data were recorded at the Vienna General Hospital, Department of Neurology, in the course of a presurgical investigation.

**Fig. 3:**
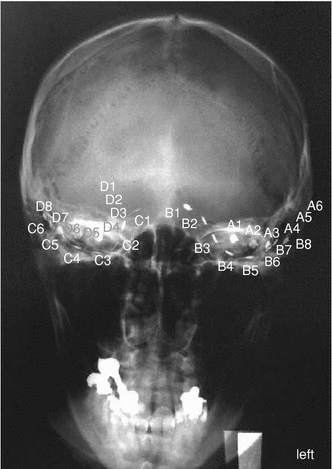
X-ray image of patient A in *frontal view* indicating electrode positions of the four strip electrodes: *A* and *B* below the left temporal lobe, *C* and *D* below the right temporal lobe

In order to test our method on neural signals, a total of 12 seizures of these patients was analyzed. Two out of four patients had to be excluded due to strong signal artifacts and spike–wave complexes during the seizures, respectively. As our method is based on the detection of synchronization, it is not suitable for the analysis of spike–wave complexes. The remaining two patients underwent presurgical evaluation for 8 (patient A) and 7 (patient B) days, respectively. For patient A, aged 59 years, ECoG was recorded from 28 electrodes grouped in 4 individual stripes. The X-ray scan of the head of patient A in Fig. 3 details the positions of the four subdural strip electrodes: Stripes A and B are situated below the left temporal lobe, C and D below the right one. In case of patient B, aged 61 years, ECoG was recorded from 32 electrodes grouped in 5 individual stripes: similar to patient A, stripes A, B, and C are situated below the left temporal lobe, D and E below the right one.

In all cases data were recorded with a sampling frequency of 256 Hz using an IT-med$^\circledR$ recording system. After recording, the ECoG data were preprocessed in Matlab$^\circledR$: Line interference was removed with a Notch filter at 50 Hz, and the signals were low-pass-filtered at 64 Hz in order to avoid aliasing and then downsampled to 128 Hz.

Table 1 summarizes the clinical findings for the seizure onset time of the two patients. This information was given by the clinicians based on a visual inspection of raw ECoG signals and will be used in Sect. 4 in order to evaluate the results of our method (cf. Sect. 3). As seizure three of patient B was primarily generalized, we do not evaluate it and thus consider six seizures in total (Table 1a–f).

Note that the recordings of patient A reveal different lateralization (cf. the X-ray image in Fig. 3): In case of seizure 1, onset of epileptic activity is on the left hemisphere, whereas in case of seizures 2, 3, and 4 the right hemisphere is initially involved.

**Table 1:** Onset zone and initial propagation of the analyzed seizures according to the visual inspection by clinicians. (a)–(d) show the clinical findings for seizures 1–4 of patient A, (e)–(g) the ones for seizures 1–3 of patient B

Time	Electrodes affected
(a) Patient A, seizure 1	
03:04:36	B1–B3
03:04:57	B1–B5
(b) Patient A, seizure 2	
12:45:51	D6, D7
12:45:52	C1–C6, D1, D4–D7
(c) Patient A, seizure 3	
12:31:41	D6, D7
12:31:52	B4, B5, D6, D7
(d) Patient A, seizure 4	
15:21:42	C1
15:21:47	C1, D4–D7
(e) Patient B, seizure 1	
07:02:02	E1, E2
07:02:03	D1, D2, E1, E2
(f) Patient B, seizure 2	
08:06:34	E1, E2
08:06:35	D1, D2, E1, E2
(g) Patient B, seizure 3	
08:55:41	Immediately generalized

**Table 2:** Values of EIPR for signal model (13). Imposed dependencies (bold values) are correctly recognized, 99 %-significance thresholds are indicated in italic between brackets

$\eta _{k,l}^{2}$	$x_{1}$	$x_{2}$	$x_{3}$	$x_{4}$
$x_{1}$	1.00000	**0.18039** (*0.05877*)	0.00005 (*0.00126*)	0.00001 (*0.00194*)
$x_{2}$	0.00066 (*0.00141*)	1.00000	0.00064 (*0.00090*)	**0.75701** (*0.24812*)
$x_{3}$	**2.09681** (*0.12395*)	**0.26936** (*0.04652*)	1.00000	0.00003 (*0.00133*)
$x_{4}$	0.00019 (*0.00031*)	0.00008 (*0.00017*)	0.00014 (*0.00025*)	1.00000

## Results

### Signal model validation by EIPR

In order to show the ability of our method to detect dependencies, we first apply EIPR to the AR model (13)^3^. Here, we disable the automatic channel selection algorithm described in Sect. 2.4 in order to assure that the entire coupling information contained in the multichannel signal is used.

We compare our findings to the result of PDC as reported by Winterhalder et al. (2005). For each source channel $x_{l}$ and target channel $x_{k}$ with $k\ne l$, Winterhalder et al. (2005) show a frequency plot of PDC $\pi _{k,l}^{2}(f)$. These frequency plots are arranged in a $K\times K$-matrix plot, where the columns indicate the source channels and the rows the target channels (compare Fig. 5 in the discussion). Thus, the (*k*, *l*)-subplot quantifies the influence from $x_{l}$ to $x_{k}$. If $\pi _{k,l}^{2}(f)=0\,\forall f$, one can conclude that there is no direct dependency from $x_{l}$ to $x_{k}$. However, as it is unlikely in applications that one observes values of PDC exactly matching zero for all frequencies, one has to use a statistical test. Thus, Schelter et al. (2005) derived an asymptotic frequency-dependent confidence interval: For each frequency $f$, PDC values below the respective threshold indicate the absence of any direct coupling.

**Fig. 4:**
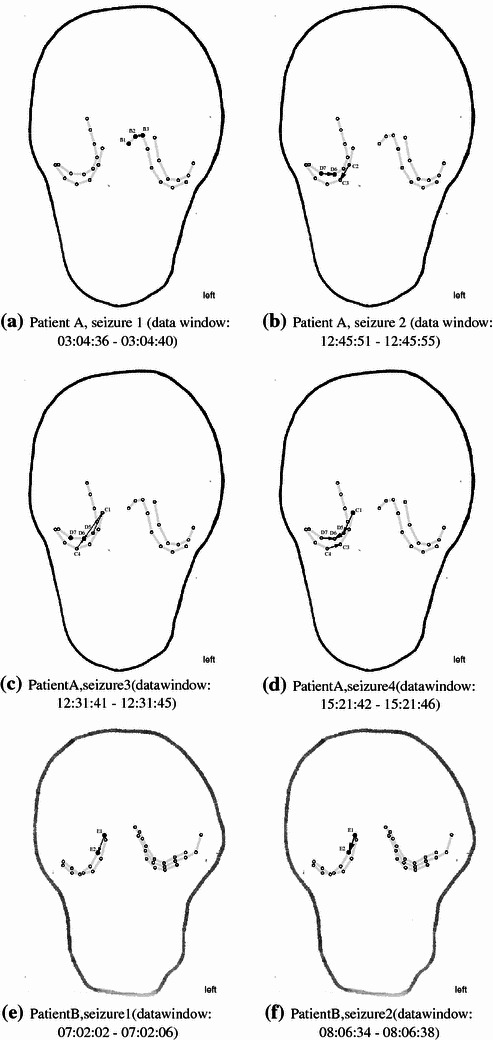
Couplings at time of seizure onset in patients A and B. Each plot represents the *frontal view* onto the patient’s head according to the surgeon’s draft. **a**–**d** represent the four seizures of patient A at the respective time of seizure onset, plots (**e**), (**f**) two seizures of patient B. In each illustration, the *thick line* symbolizes the cranium, and implanted electrode *stripes* are indicated in *light gray color*. *Circles* represent the corresponding electrode positions, *arrows* indicate high EIPR values. Electrodes which are part of the seizure onset zone according to Table 1 are represented by *filled circles*. The areas of highest EIPR values coincide well with the respective seizure onset zones

**Fig. 5:**
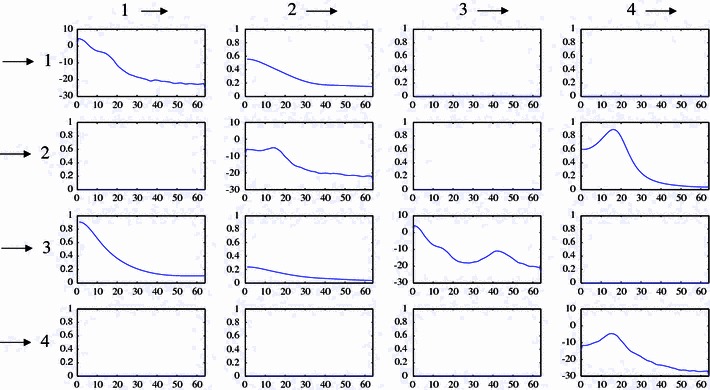
PDC matrix plot of the signal model (13), confer (Winterhalder et al. 2005). The plots on the *diagonal* show the spectra of the respective channels (*x*-axis: frequency in Hz, *y*-axis: spectrum in dB scale). A subplot on position $(k,l),\, k\ne l$ (*x*-axis: frequency in Hz, *y*-axis: PDC) visualizes the influence of $x_{l}$ to $x_{k}$ measured by $\pi _{k,l}^{2}(f)$. The imposed dependencies are correctly identified

In contrast to PDC, EIPR condenses the coupling information from $x_{l}$ to $x_{k}$ in one scalar value. Therefore, the coupling information [EIPR and significance threshold (14)] can be represented in a table: In complete analogy to PDC, the (*k*, *l*)-element of the table quantifies the influence from $x_{l}$ to $x_{k}$, and the columns indicate the source channels, the rows the targets.

**Table 3:** Step-wise behavior of the channel selection algorithm for signal model (13). Channels with imposed dependencies are selected. Minima of the BIC values of each step are set in bold-face type for better traceability

$x_{k}$	Step	Initial regression: channels, BIC	Extended regression: additional channel, BIC	Step result
$x_{1}$	1	$\{x_{1}\}$: 0.811	$x_{2}$: $-$ **0.019**	Choose $x_{2}$
			$x_{3}$: 0.783	$\mathbb L _{1}=\{x_{2}\}$
			$x_{4}$: 0.755	
	2	$\{x_{1},x_{2}\}$: $-$ **0.019**	$x_{3}$: $-$0.016	STOP
			$x_{4}$: $-$0.016	$\mathbb L _{1}=\{x_{2}\}$
$x_{2}$	1	$\{x_{2}\}$: 0.623	$x_{1}$: 0.624	Choose $x_{4}$
			$x_{3}$: 0.625	$\mathbb L _{2}=\{x_{4}\}$
			$x_{4}$: **0.011**	
	2	$\{x_{2},x_{4}\}$: **0.011**	$x_{1}$: 0.015	STOP
			$x_{3}$: 0.015	$\mathbb L _{2}=\{x_{4}\}$
$x_{3}$	1	$\{x_{3}\}$: 1.165	$x_{1}$: **0.377**	Choose $x_{1}$
			$x_{2}$: 0.592	$\mathbb L _{3}=\{x_{1}\}$
			$x_{4}$: 1.143	
	2	$\{x_{3},x_{1}\}$: 0.377	$x_{2}$: **0.001**	Choose $x_{2}$
			$x_{4}$: 0.348	$\mathbb L _{3}=\{x_{1},x_{2}\}$
	3	$\{x_{3},x_{1},x_{2}\}$: **0.001**	$x_{4}$: 0.005	STOP
				$$\mathbb L _{3}=\{x_{1},x_{2}\}$$
$x_{4}$	1	$\{x_{4}\}$: **0.014**	$x_{1}$: 0.018	STOP
			$x_{2}$: 0.018	$\mathbb L _{4}=\{\}$
			$x_{3}$: 0.018	

As detailed in Table 2, which is constructed in this way, our measure correctly identifies the imposed dependencies illustrated in Fig. 2b: The ones which are induced by the signal model (13) are set in bold-face type. Similar to PDC, we do not expect to observe EIPR values exactly matching zero in case of non-causality. We rather have to decide whether EIPR $\eta _{k,l}^{2}$ significantly differs from zero by exceeding 99 % significance thresholds (detailed in italic between brackets behind the respective EIPR values in Table 2).

### Analysis of the channel selection algorithm

In a next step, we analyze the dynamical channel selection algorithm by applying it to the AR model (13) without subsequent calculation of EIPR. As stated in Sect. 2.4, we expect our algorithm to select the extrinsic channels which contribute significantly to the explanation of the respective intrinsic channel. The simple structure of signal model (13) allows to verify this design: In this artificial case, the imposed signal model dependencies (cf. Fig. 2b) exhaustively define the important extrinsic channels for each intrinsic one. Unlike in the case of ECoG recordings, we do not have any additional weak dependencies here which we want to single out for numerical reasons.

Table 3 illustrates the results of this simulation. As expected, the algorithm builds up the extrinsic channels sets in accordance with the imposed dependencies.

First, consider channel $x_{1}$, which is only influenced by $x_{2}$. The algorithm sets $\mathbb L _{1}=\{x_{2}\}$, as in the first step the BIC value of the extended regression using channels $x_{1}$ and $x_{2}$ is minimal. In the second step, a further increase of the number of regressors does not lead to a decrease of the information criterion any more, and the algorithm stops returning $\mathbb L _{1}=\{x_{2}\}$.

Next, consider channel $x_{2}$. Similarly to the previous case, it is only influenced by one channel, namely $x_{4}$, and we obtain $\mathbb L _{2}=\{x_{4}\}$.

The situation is different in case of channel $x_{3}$ which is influenced by $x_{1}$ as well $x_{2}$. In a first step, the algorithm selects the channel with the strongest influence [AR coefficient of $-$0.6, cf. model (13)], $x_{1}$. In a second step, $x_{2}$ is chosen (AR coefficient of 0.4). In a third step, the information criterion cannot be reduced, and the algorithm stops returning $\mathbb L _{3}=\{x_{1},x_{2}\}$.

Finally, we obtain an empty extrinsic channel set for $x_{4}$, as the regression based on $x_{4}$ alone minimizes BIC.

### Application to neural data

We apply our proposed methodology, i.e., regression with dynamically selected channels and subsequent calculation of EIPR, to ECoG recordings from epilepsy patients in order to localize the seizure onset zone. Its identification is based on the analysis of the dependency measure calculated in the initial seconds of the seizure, given the exact seizure onset time (cf. Sect. 2.9).

The ECoG data used for this purpose were acquired as described in Sect. 2.9 and include a total number of six epileptic seizures, four of patient A and two of patient B. Data were processed within windows of 4 s, as this value turned out to be a good trade-off for the estimation quality of the correlation matrix between estimation errors due to instationarity and inaccuracy due to a too small number of samples. An AR model order of $p=7$ was chosen. This allows for the modeling of a spectrum with three peaks (e.g., in the $\vartheta$-band and two others) and slowly changing components (Marple 1987). This choice is in good accordance with simulations yielding the optimal model order (Graef 2008).

The results obtained with EIPR within the first window after seizure onset are illustrated in Fig. 4. In each subplot, the frontal view onto the patient’s head is symbolized (compare Fig. 3 for the corresponding X-ray scan of patient A). In each of these six illustrations, the cranium is symbolized by a thick line according to the surgeon’s draft. Light gray lines indicate the implanted subdural stripes, and circles represent the exact electrode positions. Electrodes which belong to the seizure onset zone according to the visual inspection are symbolized by filled circles. For easier comparison of Fig. 4 with the clinical findings detailed in Table 1, important electrodes are labeled with their respective identifier.

Plots (a)–(d) represent the four seizures of patient A at the respective time of seizure onset, plots (e)–(f) two seizures of patient B. An arrow from electrode $x_{l}$ to electrode $x_{k}$ indicates that $\eta _{k,l}^{2}$ exceeds a threshold, i.e., indicates strong direct coupling from $x_{l}$ to $x_{k}$. These two electrodes are associated with an area of increased coupling activity.

For patient A, the indicated areas comprise electrodes on both hemispheres. In case of seizure 1 (plot a), the electrodes B2 and B3 show the highest EIPR on the left hemisphere. At the onset time of the three subsequent seizures, the respective area of highest EIPR values is located on the right hemisphere: electrodes C2, C3, D6, and D7 for seizure 2 (plot b); C1, C4, D5, and D6 for seizure 3 (plot c); C1, C3, C4, D5, D6, and D7 for seizure 4 (plot d). As for patient B, EIPR identifies the electrodes E1 and E2 on the right hemisphere for seizures 1 (plot e) as well as 2 (plot f). The third seizure of patient B is not shown, as it immediately generalizes (cf. Sect. 2.9).

For both patients, the areas of increased coupling activity as indicated by EIPR coincide well with the respective seizure onset zones specified by the clinicians (compare Table 1). In case of patient A, our results are in good accordance with the visual inspection detailed in Table 1. Note that our methodology correctly indicates the increased couplings on the left hemisphere for the first seizure and on the right hemisphere for the three subsequent seizures. Thus EIPR correctly identifies the different lateralization of the initial epileptic activity in patient A. Considering patient B, our results are in very good accordance with the clinical findings. For both seizures, we identify increased couplings between the two electrodes which show initial epileptic activity according to the visual inspection.

## Discussion

### EIPR as coupling indicator

In this paper, we introduce a novel approach to the quantification of directed couplings. The proposed dependency measure EIPR indicates Granger causality (as does the PDC), but has the advantage of a clear physical interpretation as a power ratio, compare expression (6). As mentioned in Sect. 2.6, its normalization assures that the measured coupling strength between two channels is not influenced by others. This behavior is in contrast to the one of PDC (Graef et al. 2009) whose confidence level depends on all neighborhood channels in order to compensate for the normalization effect (Schelter et al. 2005).

Due to its construction, EIPR successfully validates the signal model (13). It retrieves the imposed dependencies (compare Table 2), as does PDC (compare Fig. 5). In both cases, the couplings $x_{1}\rightarrow x_{3}$ (position (3,1) in the scalar matrix / matrix plot) and $x_{4}\rightarrow x_{2}$ (position (2,4) in the scalar matrix / matrix plot) are predominantly indicated.

Here, we want to discuss three additional aspects regarding the comparison of EIPR and PDC.

First, we observe an interesting behavior of EIPR: The statistically significant values in Table 2 exceed the non-significant ones by a factor of 100. Even at a first glance at such an EIPR table (without comparing the EIPR values to their respective significance thresholds) we would obtain an idea about the underlying dependence structure. Note that the PDC matrix plot in Fig. 5 creates a similar impression, but in case of EIPR the tendency to separate significant from non-significant values is stronger. This is a result of the normalization discussed above.

Second, the EIPR values in Table 2 range between 0 and 2, PDC is normalized between 0 and 1. The reason for the scatter of the EIPR values is the following: The variance of the extrinsic contribution term in the nominator of EIPR represents the power of the extrinsic contribution, which is the integral of the corresponding power spectral density over all frequencies [compare expression (11)]. In the nominator of PDC the same integrand shows up, but for a single frequency [compare expression (12)]. Therefore, EIPR takes large values for couplings where PDC is increased over a wide frequency range, compare Fig. 5. In particular, this is the case for the two couplings mentioned above, $x_{1}\rightarrow x_{3}$ and $x_{4}\rightarrow x_{2}$. Vice versa, PDC vanishing over a large frequency band results in very small EIPR values (e.g., coupling $x_{3}\rightarrow x_{1}$).

**Fig. 6:**
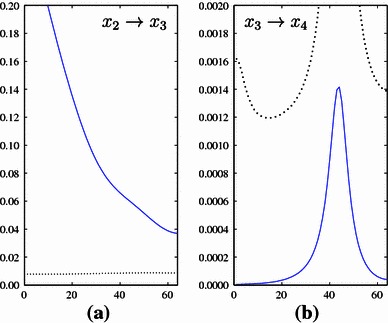
Zoom into two subplots of the PDC matrix plot in Fig. 5 (*x*-axis: frequency in Hz, *y*-axis: PDC). **a** zoom of the subplot on position (3,2) indicating the dependence $x_{2}\rightarrow x_{3}$, **b** zoom of the subplot on position (4,3) indicating the dependence $x_{3}\rightarrow x_{4}$. PDC values are illustrated by *solid lines*, significance thresholds by *dotted lines*. For each plot, a different zoom factor is necessary to allow for a simultaneous visualization of PDC values and significance thresholds. A direct comparison between the different plots is difficult

Third, an advantage of EIPR is its compact representation in the form of a matrix of (physically meaningful) scalar values as in Table 2. This allows for a simultaneous comparison of the individual EIPR values with their respective significance thresholds even in case of large scale differences. In contrast, a PDC matrix representation has the drawback of being difficult to interpret. One has to consider the respective subplot and compare PDC to the significance threshold for all frequency points. However, as mentioned in Schelter et al. (2005), this point-wise comparison is not straight-forward. Consider for example the significant couplings $x_{2}\rightarrow x_{3}$ and the non-significant ones $x_{3}\rightarrow x_{4}$ for both measures. Comparing the EIPR values $\eta _{3,2}^{2}$ and $\eta _{4,3}^{2}$ with their respective significance thresholds is easily performed in Table 2. In case of the PDC matrix plot in Fig. 5, this simple evaluation is not possible. Small PDC values and significance thresholds are not easily visible due the large scale differences. In order to allow for a clear visualization of the PDC values and their thresholds in each subplot (simultaneously visible), each subplot would have to be scaled differently. Compare Fig. 6 for an illustration, where the scaling of the two subplots of couplings $x_{2}\rightarrow x_{3}$ and $x_{3}\rightarrow x_{4}$ is performed in this way. Here, the PDC values and significance thresholds of both couplings are visible, at the price of a scale difference of factor 100. This would render the comparison of the PDC values between different subplots in a matrix plot such as Fig. 5 difficult.

We want to conclude this part of the discussion with two comments on the interpretation of EIPR.

EIPR is not normalized between 0 and 1, which is a drawback in comparison to PDC. In particular, this impairs the comparison between different systems, as equal EIPR values might not indicate the same coupling strength in distinct multichannel signals.

On the other hand, EIPR allows for an interpretation similar to the signal-to-noise ratio (SNR): Given a signal $x[n]=u[n]+z[n]$ consisting of meaningful information $u[n]$ and background noise $z[n]$, the SNR is commonly defined (using the logarithmic dB scale) as

$$\begin{aligned} \text{ SNR} \triangleq 10 \, \lg \left(\frac{\mathbb{V }\left\{ u[n]\right\} }{\mathbb{V }\left\{ z[n]\right\} }\right). \end{aligned}$$

Thus EIPR can be intuitively interpreted: The extrinsic contribution takes the roles of the information we are interested in, and the intrinsic contribution is seen as background noise. This interpretation underlines the influence of the extrinsic information for quantifying the coupling strength, which is in particular important in the dependence analysis of epileptic ECoG recordings. We will discuss this setting in Sect. 4.3.

### Behavior of the channel selection algorithm

As demonstrated in Sect. 3.2, the dynamic channel selection algorithm behaves as expected in simulations, selecting channels with influence and discarding the others: Applied to the signal model (13), it builds up the respective extrinsic channel sets in accordance with the dependencies imposed. Moreover, it is capable of prioritizing extrinsic channels with strongest influence.

This behavior strengthens our hypothesis that the algorithm performs well in ECoG data: Here, we encounter many influences with few important ones (representing epileptic activity): The order according to which the extrinsic channel set is built up is important, as the proposed forward-selection procedure does not search through the whole parameter space. Due to the simulation results discussed above, we are confident that the algorithm’s focus on strongest influence selects the important channels first, thus including the channels of interest in the extrinsic channel set. This assures that EIPR can be calculated and visualized between highly coupled channels in the subsequent step.

### Seizure onset zone localization

In this paper, we assume that the area of highest EIPR values in the initial seconds after seizure onset indicates the seizure onset zone. Our reasoning is the following: As mentioned in Sect. 1.1, in case of focal epilepsy the pathological synchronous activity starts at a small localized brain area. Departing from this seizure onset zone it spreads to its immediate vicinity recruiting more and more parts of the neural network. This leads to a hyper-synchronous behavior of the observed channels. One could imagine a “focus” located in the seizure onset zone driving the surrounding channels by imposing its oscillatory frequency in the course of the recruiting process. This could be interpreted as a kind of information transfer: Imagine one electrode in the focus, say $x_{1}$, influencing the behavior of the surrounding electrodes, say $x_{2}$ and $x_{3}$, in the initial phase of the seizure. Then, sticking to this image of information transfer, we expect the extrinsic contributions from $x_{1}$ to $x_{2}$ and $x_{3}$ to show high values and the intrinsic contribution terms of $x_{2}$ and $x_{3}$ to be small. This results in high EIPR values $\eta _{1,2}^{2}$ and $\eta _{1,3}^{2}$, we observe increased directed coupling activity symbolized by arrows. By limiting our representation to the highest EIPR values within each analysis, we focus on these pathological synchronizations and do not regard others (e.g., weak physiological ones).

In the course of the recruiting process, we obviously expect feedback mechanisms within the seizure onset zone due to the excessive synchronous activity. Therefore, we expect to see two different kinds of arrows: Besides unidirectional information out-flow from the seizure onset zone into the surrounding areas we will typically observe arrows pointing from one electrode to another within the seizure onset zone. In both cases, the associated electrodes are the ones we aim to identify.

We believe that the results presented in Sect. 3.3 strengthen this hypothesis and would like to discuss two aspects regarding the quality of our findings.

The first interesting aspect is the indicated direction of information flow. Consider seizure 4 of patient A in Fig. 4d. We observe a clear path of information flow departing from the electrode C1 (marked as seizure onset zone by the clinicians) to D5 and onwards to D6. In plots (a), (b), (e), and (f), we encounter the second type of expected arrows, information flow within the seizure onset zone. These cases exactly meet our expectation. However, in plot (c) the direction of information flow is opposite. An arrow points from C1 to the seizure onset zone, which is due to the fact that electrode C1 shows strong anteceding rhythmic activity. This pattern was not classified as epileptic by the clinicians but contributes to the explanation of D6 in the regression model.

The second aspect we want to mention is the temporal delay between EIPR indications and clinical findings. Consider seizure 2 of patient A in Fig. 4d, which shows coupling activity between D6 and D7 and C2 and C3. In this case, EIPR indicates the seizure onset zone as well as the initial subsequent propagation: According to Table 1, the seizure onset zone comprises electrodes D6 and D7 at 12:45:51, but the synchronization swaps over to others within 1 s (12:45:52). As the employed data window of 4 s comprises data from 12:45:51 to 12:45:55, these fast spectral changes influence the regression. The situation is slightly different in the case of seizure 4 of patient A in plot (d). The seizure onset zone comprises the electrode C1 on the right hemisphere at 15:21:42, which is correctly indicated by the EIPR. However, the electrodes D5, D6, and D7 are also highlighted, but are only affected 5 s after seizure onset (15:21:47). We thus observe an anticipation of 5 s, which is outside the employed window of 4 s. Thus, the question arises whether EIPR might be able to identify slow spectral changes of the signal which are difficult to detect by visual analysis of the raw ECoG recordings.

In all investigated seizures, the indicated area of highest EIPR values is well correlated with the seizure onset zone as indicated by the clinicians. In particular, we are able to track the different lateralization of patient A (onset of seizure 1 on the left hemisphere, onset of seizures 2, 3, and 4 on the right hemisphere). We would like to point out that in these cases the achieved spatial accuracy of our method is in the range within 1–2 cm (the distance between the electrodes is ${\sim }$1 cm, compare the X-ray image in Fig. 3).

### Concluding remarks

In this paper, we proposed a novel dependency measure which is capable of reliably measuring coupling effects in multivariate signals as well as an automatic channel selection algorithm. In particular, we are able to identify synchronization effects in ictal multichannel ECoG recordings which allows us to draw conclusion on the localization of the seizure onset zone in the given examples. We want to conclude this discussion with two side-remarks detailing alternatives.

First, the temporal lag order of the regression is kept constant. As a potential drawback, this might lead to under- or over-fitting of the AR model and consequent erroneous dependencies. In order to avoid this situation one could extend the regression to a model with variable temporal lag order [determined by means of an extended version of the BIC criterion (4)]. In this case, the model would better reflect the spectral properties of the EEG (Marple 1987), but at the price of a higher computational effort: As a function of the data-driven model order a dynamic window length (which is currently fixed) would have to be defined such that a sufficiently reliable estimation of the necessary AR parameters is possible.

Second, in this paper we use a dynamic channel selection algorithm (cf. Fig. 1) to overcome the estimation issues due to the high correlation in the cross-sectional dimension. Another approach which might be appropriate for this task is penalized regression, e.g., LASSO as introduced by Tibshirani (1996). Here, one is confronted with the solution of a convex problem instead of the linear normal equations. Only recently, Chiang et al. (2009) successfully applied this approach to neural data, calculated PDC, and visualized the indicated brain connectivity of participants taking part in a virtual-reality experiment.

Concluding, we believe that the aspects discussed in this section strengthen our hypothesis of EIPR being a useful measure for the characterization of neurophysiological dependencies. Therefore, we think that our methodology has the potential to assist clinicians in the presurgical evaluation of epilepsy patients by objectivating the visual ECoG examination: Tracking the synchronization effects over time might indicate the seizure onset zone as well as the initial propagation of the epileptic activity.

## Footnotes

1 As the well-known *Akaike information criterion* (AIC) tends to overfit, we use BIC introduced by Schwarz (1978). Statistical properties are discussed in Hannan and Deistler (1988).

2 The case of $\mathbb{V }\left\{ \mu _{k,k}[n]\right\} =0$ would imply that the past of $x_{k}$ does not contribute to the explanation of $x_{k}$ in the present, i.e., the chosen $x_{l}$ (alone) explain $x_{k}$ optimally. This is highly unlikely in ECoG data, which was empirically shown in (Graef 2008).

3 Simulation of this model was done with the help of the Matlab$^\circledR$ package ARFIT developed by Schneider and Neumaier (2001). In order to avoid initial transient effects, we generate 13,800 samples and discard the first 1,000.
